# Ethyl 3-mercaptopropionate, a safe food flavoring, competitively inhibits polyphenol oxidase and prevents browning in fresh-cut produce

**DOI:** 10.1016/j.fochx.2025.102859

**Published:** 2025-08-02

**Authors:** Guangcan Cui, Juncang Peng, PingPing Liu, Yonghong Wang, Chang Ge, Xiaoyong Chang, Xueao Zheng, Chen Wang, Yalong Xu, Xiaozhan Qu, Yixiao Zhang, Peijian Cao, Tengfei Liu, Qiansi Chen

**Affiliations:** a Zhengzhou Tobacco Research Institute of CNTC, No. 2 Fengyang Street, Zhengzhou, Henan Province 450001, China; b College of Food Science and Engineering, Shandong Agricultural University, Tai'an 271018, Shandong, China; c Technology Center, China Tobacco Shaanxi Industrial Co., Ltd., Xian 710065, China; d Beijing Life Science Academy (BLSA), Beijing 102209, China

**Keywords:** Polyphenol oxidase, Ethyl 3-Mercaptopropionate, Fresh-cut, Browning, Molecular dynamics simulations

## Abstract

Enzymatic browning, caused by polyphenol oxidase (PPO), degrades fresh-cut produce, resulting in significant economic losses. This study investigates ethyl 3-mercaptopropionate (EMP), a safe food flavoring, as a novel PPO inhibitor. EMP at 50–100 μL/L effectively prevented browning in both fresh-cut potatoes and tobacco leaf pulp, performing comparably to the standard inhibitor sodium bisulfite but offering better safety. Purified potato PPO showed nanomolar sensitivity to EMP with an IC₅₀ of 156.7 ± 17.26 nM. Molecular modeling and dynamics simulations revealed that EMP acts as a competitive inhibitor, competing with the natural substrate for the PPO active site. By binding key active site residues, including copper-coordinating histidines, EMP induces a more rigid enzyme structure, hindering its catalytic activity. These findings establish EMP as a potent competitive inhibitor, presenting a promising, safe, and effective strategy to control enzymatic browning in fresh-cut produce. This research also provides valuable mechanistic insights for developing improved anti-browning methods.

## Highlights

• Ethyl 3-mercaptopropionate (EMP) prevents enzymatic browning in fresh-cut potatoes.
• EMP potently inhibits PPO activity with an nM-level IC₅₀ in potato PPO.
• Molecular modeling shows EMP competitively binds PPO, displacing 4-MC from the active site.
• As a safe flavoring, EMP provides a superior alternative to sulfites for browning control.

## 1 Introduction

Enzymatic browning is a major cause of quality deterioration in fresh and minimally processed fruits and vegetables, leading to significant economic losses in the food industry (Moon et al., 2020). This undesirable phenomenon is primarily catalyzed by polyphenol oxidase (PPO), a copper-containing enzyme that oxidizes phenolic compounds to *o*-quinones (Sui et al., 2023). These quinones subsequently undergo non-enzymatic polymerization reactions, forming complex brown pigments known as melanins (Tilley et al., 2023). The resulting discoloration negatively affects the visual appearance, consumer acceptability, and market value of fresh-cut produce (Li et al., 2025; Xiao et al., 2021).

Potatoes (*Solanum tuberosum* L.) are a globally important staple crop and are highly susceptible to enzymatic browning upon peeling, cutting, or other tissue damage (Liu et al., 2023; Tsikrika et al., 2022). This susceptibility is primarily attributed to their high levels of phenolic substrates and active PPO (Li et al., 2023). Browning in fresh-cut potatoes impacts their aesthetic appeal and can change flavor, texture, and nutritional value (Zhang, Fu, et al., 2025). Therefore, effective strategies to control enzymatic browning are crucial for maintaining the quality and extending the shelf-life of fresh-cut potato products.

Various physical and chemical methods have been employed to inhibit PPO activity and mitigate browning. Physical methods include heat treatment, modified atmosphere packaging, and irradiation (Liang et al., 2024; Navina et al., 2023; Xu et al., 2022). However, these methods can sometimes have detrimental effects on the sensory and nutritional properties of the produce. Chemical methods often involve anti-browning agents that inhibit PPO activity, reduce *o*-quinones, or chelate copper ions (Fan, 2023; Ma et al., 2021). Sulfiting agents, exemplified by sodium bisulfite, are potent inhibitors of enzymatic browning commonly utilized in food processing (Nath et al., 2022). Other chemical inhibitors, such as ascorbic acid and sodium isoascorbate, demonstrate notable efficacy in preventing browning reactions (Xiong et al., 2025). Despite their efficacy, concerns have arisen regarding their potential to elicit adverse health reactions, especially allergic responses in sensitive populations (Iyengar & McEvily, 1992). Consequently, there is a growing impetus to develop and implement alternative strategies for controlling enzymatic browning that do not pose the same health risks.

Recent research has focused on natural compounds as safe alternatives to sulfites for browning control (Khan et al., 2021; Zhang et al., 2022; Zhang, Wang, et al., 2025). Notable examples include ursolic acid, which effectively inhibits PPO in fresh-cut apples through competitive binding mechanisms (Zhang, Wang, et al., 2025), and oxyresveratrol from white mulberry bark, which demonstrates substantial anti-browning efficacy in potato products (Zhang, Fu, et al., 2025). Similarly, chlorogenic acid suppresses surface discoloration while enhancing antioxidative capacity (You et al., 2024). These natural compounds offer advantages in consumer acceptance, environmental sustainability, and reduced health risks compared to synthetic inhibitors.

Previous research has conclusively identified ethyl-3-mercaptopropionate (EMP) as a naturally occurring volatile constituent of *Vitis labrusca* (Concord) grapes, making substantial contributions to their characteristic musky aroma, often described as “foxy” (Kolor, 1983). Subsequent research on the volatile profiles of ripened cheeses has established EMP as a potent aroma-active compound in both Munster and Camembert, playing a pivotal role in the ontogeny of their distinctive flavor profiles (Sourabié et al., 2008). EMP is recognized as safe by the European Union (EU) and the Joint FAO/WHO Expert Committee on Food Additives (JECFA), being included on the EU's authorized flavoring substances list and having undergone successful JECFA safety evaluations, thus confirming its suitability for food use. Its established safety as a flavor renders it a promising candidate for further investigation into other food applications, including its potential as an anti-browning agent.

EMP features a thiol moiety, a structural element common to several naturally occurring compounds with recognized antioxidant and anti-browning properties. Notably, sulfur-containing amino acid cysteine has been reported to inhibit polyphenol oxidase (PPO) activity (Li et al., 2024; Zhang et al., 2023). Similarly, specific volatile sulfur compounds in *Allium* species, including garlic and onion, exhibit anti-browning properties (Bobo et al., 2022; Kim et al., 2005; Liang et al., 2024; Zhang et al., 2022). These findings suggest that sulfur-containing compounds, particularly those with thiol groups like EMP, may possess the capacity to inhibit PPO and mitigate enzymatic browning.

Therefore, the specific objectives of this study were to: (1) evaluate the efficacy of EMP as an anti-browning agent in fresh-cut potatoes at various concentrations; (2) elucidate the mechanistic basis of EMP's inhibitory action through enzyme kinetics and molecular modeling; (3) investigate the competitive inhibition mechanism using computational approaches; and (4) assess the impact of EMP treatment on the quality attributes of fresh-cut potatoes during storage. The findings will contribute to developing efficacious and safe strategies for controlling enzymatic browning in fresh-cut produce, potentially mitigating food waste, enhancing product quality, and increasing consumer satisfaction. Given EMP's established safety as a flavor, it presents a promising alternative to conventional anti-browning agents, such as sulfites, offering a valuable tool for the food industry. Furthermore, this research will elucidate the molecular interactions between EMP and PPO, providing insights into the rational design of novel, more potent PPO inhibitors.

## 2 Materials and methods

### 2.1 Materials and treatments

Potatoes (*Solanum tuberosum* L., cv. ‘Netherlands 15’) were purchased from a local vegetable market in Taian, China. Tubers with uniform shape and free from disease and mechanical damage were selected and stored in cardboard boxes lined with polyethylene (PE) bags at 2–4 °C until further use.

Ethyl 3-mercaptopropionate (EMP) reagent was purchased from Maclean Biochemical Technology Co., Ltd. (Shanghai, China). EMP solutions were prepared at concentrations of 0 (Control), 10, 25, 50, 75, and 100 μL/L, each containing 0.5 % ethanol. Sensory evaluation of treatment solutions revealed that EMP at concentrations up to 100 μL/L imparted a mild, fruity‑sulfur aroma characteristic of its natural occurrence in Concord grapes and ripened cheeses. This aroma was barely perceptible in treated potato samples after draining and was not detected by panelists during storage. A 0.25 % (*w*/*v*) sodium bisulfite solution was prepared using deionized water. This concentration was selected as a positive control based on its established efficacy as a browning inhibitor in previous studies (Ge et al., 2025)*.*

Eighty potato tubers were washed with tap water. After peeling, the potatoes were cut into approximately 7–8 cm in length and 2–3 mm in width and thickness. The fresh-cut potato shreds were mixed thoroughly. Fresh-cut shreds were immersed in each prepared solution for 20 min, drained using cheesecloth, and packaged in PE bags (approximately 100 g per bag, 15 bags per treatment). The bags were folded at the top and stored at 4 °C. On days 0, 1, 2, 3, and 4, colorimetric measurements and visual evaluations were performed on all fresh-cut potato shreds, using three bags of shreds per treatment for sensory quality assessment.

### 2.2 L* value, visual browning index, and visual quality assessment

Lightness (L*) values were measured using a CR-400 colorimeter (Minolta Co., Osaka, Japan) calibrated with a white standard (L* = 97.06, a* = 0.04, b* = 2.01). For each treatment and time point, measurements were taken on 30 randomly selected potato shreds from three replicate bags. Visual browning was evaluated by a six-member trained panel under standardized conditions (D65 illumination, 6500 K, neutral gray backdrop) using a 5-point scale: 1 = no browning (0 % affected area), 2 = trace browning (≤5 %), 3 = moderate browning (5–20 %), 4 = marked browning (20–50 %), and 5 = extensive browning (>50 %). Panelists assessed samples from three bags per treatment/time point, with final scores determined by consensus or averaging. The overall visual quality was assessed using a 9-point hedonic scale (1 = extremely unacceptable, 9 = excellent), with a focus on color uniformity, absence of browning, surface freshness, and visual appeal. Composite quality scores were calculated as Σ (individual score × proportion of shreds at that score).

### 2.3 The impact of EMP on pH values, tissue pulp preparation, and EMP treatment for browning assays

To assess the potential impact of EMP on solution pH values, pH measurements of the 0 μL/L (Control) and 50 μL/L EMP solutions were performed using a pH meter (S40 Seven, Mettler Toledo, Shanghai, China).

Homogenates of potato tuber and tobacco leaf were prepared for browning inhibition studies. Uniform and undamaged potato tubers and four-week-old tobacco leaves (*Nicotiana tabacum* cv. K326) were washed, peeled (potatoes), and diced. Separate pulps were created by homogenizing 5 g of each diced tissue with 10 mL of deionized water.

Potato pulp was treated with 0 (Control) and 50 μL/L EMP solutions, while tobacco pulp was treated with concentrations of 0 (Control), 10, 25, 50, 75, and 100 μL/L EMP solutions. Incubation was performed at 25 °C, with periodic sampling to determine browning. Spectrophotometric absorbance at 410 nm was used to quantify browning in the potato tuber pulp.

### 2.4 PPO activity analysis

Crude polyphenol oxidase (PPO) extract was prepared from frozen potato powder using a modified method based on Feng, Zhang, et al. (2022). A centrifuge tube combined one gram of frozen potato powder with 0.5 g of insoluble polyvinylpyrrolidone (PVPP). Phosphate buffer (2.0 mL, 0.1 M, pH 6.8) was added, and the mixture was thoroughly vortexed. Following centrifugation at 10,000 ×*g* for 10 min at 4 °C, the supernatant was collected as the crude enzyme extract. PPO activity was determined following the previous report (Ge et al., 2025).

### 2.5 Recombinant PPO expression, purification, and inhibition assay

The gene encoding a truncated form of potato polyphenol oxidase (PPO), specifically the POT32 isoform (GenBank accession number U22921.1) lacking the signal peptide (the first 96 amino acids) (Thygesen et al., 1995), was cloned into a pSMART-I vector using *Bam*HI and *Xho*I restriction sites. This vector provided an N-terminal 6 × HIS-SUMO tag for purification and solubility enhancement. The specific primers used for gene amplification and cloning are detailed in Table S1. The recombinant plasmid was transformed into chemically competent *Escherichia coli* BL21(DE3) cells for protein expression. Bacterial cultures were grown at 37 °C in Luria-Bertani (LB) medium supplemented with 50 mg/L kanamycin until the optical density at OD_600_ reached 0.6–0.8. Recombinant protein expression was induced by adding isopropyl β-D-1-thiogalactopyranoside (IPTG) to a final concentration of 0.5 mM, followed by overnight incubation at 16 °C. Cells were harvested by centrifugation (5000 ×*g*, 15 min, 4 °C) and resuspended in lysis buffer containing 50 mM Tris-HCl (pH 8.0), 300 mM NaCl, 20 mM imidazole, 1 mM phenylmethylsulfonyl fluoride (PMSF), and 1 mM dithiothreitol (DTT). Cell lysis was achieved by sonication on ice, and the lysate was clarified by centrifugation (15,000 ×*g*, 45 min, 4 °C) to remove cellular debris.

The clarified supernatant from the *E. coli* lysate was loaded onto a Ni-NTA affinity column pre-equilibrated with binding buffer (50 mM Tris-HCl pH 8.0, 300 mM NaCl, 20 mM imidazole). The column was washed with wash buffer (50 mM Tris-HCl pH 8.0, 300 mM NaCl, 40 mM imidazole) to remove unbound proteins. The 6 × HIS-SUMO-tagged PPO was eluted with elution buffer (50 mM Tris-HCl pH 8.0, 300 mM NaCl, 250 mM imidazole.

Fractions collected during the purification process were analyzed by sodium dodecyl sulfate-polyacrylamide gel electrophoresis (SDS-PAGE) under reducing conditions using a 12 % polyacrylamide gel. The samples were prepared by mixing with loading buffer and heating at 95 °C for 5 min. The GoldBand Plus 3-color Regular Range Protein Marker 8–180 kDa (Yeasen, Shanghai, China) was included for size determination. Following electrophoresis, the gel was stained with Coomassie Brilliant Blue R-250 to visualize protein bands. The fractions analyzed included the crude potato extract, the supernatant after the initial centrifugation, the flow-through from the Ni-NTA column, a wash fraction, and the eluted fraction containing the purified PPO. The expected molecular weight of the purified, truncated PPO was approximately 72 kDa.

The inhibitory effect of ethyl 3-mercaptopropionate (EMP) on purified potato PPO activity was determined using a spectrophotometric assay. The enzyme inhibition system consisted of 50 μL of purified PPO solution (0.14 mg/mL) and varying volumes (0–75 μL) of a 5 mL/L EMP solution. The volume was adjusted to 125 μL using a 0.5 % ethanol solution in water, serving as the EMP solvent. This mixture was pre-incubated at 25 °C for one hour. A 50 μL aliquot of the pre-incubated enzyme-inhibitor mixture was used for subsequent enzyme activity determination.

The percentage of remaining PPO activity was calculated relative to the control (no inhibitor). The IC_50_ value, defined as the concentration of EMP required to inhibit 50 % of PPO activity, was determined by fitting the data to a dose-response curve using GraphPad Prism 10, a non-linear regression analysis.

### 2.6 Molecular modeling and interaction analysis of potato PPO with 4-MC and EMP

Molecular interactions between potato PPO, 4-methylcatechol (4-MC), and EMP were investigated using the Chai-1 multi-modal foundation model, which demonstrates competitive performance in protein-ligand prediction and superior protein complex modeling (Discovery et al., 2024). The POT32 active domain sequence (residues Pro127-Lys440) was submitted to Chai-1 via its web interface (https://lab.chaidiscovery.com/dashboard). Input parameters were protein type with 1 copy, two Cu^2+^ ions as ligands ([Cu + 2]), and ligand SMILES strings for 4-MC (PubChem CID: 9958) and EMP (PubChem CID: 21625). Three complexes were modeled: PPO-4-MC, PPO-EMP, and ternary PPO-4-MC-EMP, each with appropriate ligand copy numbers specified. Model reliability was assessed using domain-specific metrics, including RMSD, DockQ, and LDDT scores provided by Chai-1. Predicted structures were visualized using ChimeraX v.1.9 (Meng et al., 2023), and intermolecular interactions were analyzed using LigPlot^+^ v.2.2 (Laskowski & Swindells, 2011) to identify hydrogen bonds, hydrophobic contacts, and metal coordination.

### 2.7 Molecular dynamics simulation

Molecular dynamics (MD) simulations were performed on the predicted complex structures obtained from Chai-1, employing the GROMACS 2024.3 package (Abraham et al., 2015). Simulations were carried out under conditions mimicking a physiological environment: a temperature of 300 K and a pressure of 1 bar. Intermolecular forces were described using the AMBER99SB-ILDN force field, and the TIP3P model was used for explicit water solvation. Before simulations, each system was neutralized by the addition of chloride ions. The simulation protocol encompassed three stages: (1) an initial energy minimization to remove unfavorable steric contacts and optimize the system's geometry; (2) a two-stage equilibration, first in the NVT ensemble (100 ps) followed by the NPT ensemble (100 ps), both with a coupling constant of 0.1 ps; and (3) a 100 ns production run using a 2 fs integration time step.

### 2.8 Determination of dry matter content, soluble solids content, and textural hardness

Dry matter content was determined using the oven-drying method. For each treatment, three samples (approximately 5 g each) were randomly selected from each of three replicate bags. Samples were weighed (initial weight, W₁) and placed in pre-dried, pre-weighed aluminum dishes. The samples were then dried in a forced-air oven at 105 °C for 24 h until constant weight was achieved. After drying, samples were cooled in a desiccator for 30 min and reweighed (final weight, W₂). Dry matter content was calculated using the following equation:

$$\mathrm{Dry}\;\text{matter content}\;\left(\%\right)=\left(W₂/W₁\right)\times 100$$

Soluble solids content (SSC) was measured using a digital refractometer. Three potato samples were randomly selected from each of three replicate bags for each treatment. The samples were finely chopped and pressed using a garlic press to extract juice. A few drops of the extracted juice were placed on the prism surface of the refractometer, and SSC readings were recorded as degrees Brix (°Brix), representing the percentage of soluble solids by weight. All measurements were conducted at room temperature (25 ± 2 °C). Textural hardness was determined following the previous report (Cui et al., 2024).

### 2.9 Statistical analysis

A completely randomized experimental design was employed, with at least three biological replicates per treatment. Data are presented as mean values ± standard deviation. Statistical analyses were performed using analysis of variance (ANOVA), and post hoc comparisons were made using Tukey's HSD test when appropriate. Pairwise comparisons were conducted using Student's *t*-tests.

## 3 Results

### 3.1 EMP effectively inhibits browning and maintains quality in fresh-cut potatoes

**Fig. 1:**
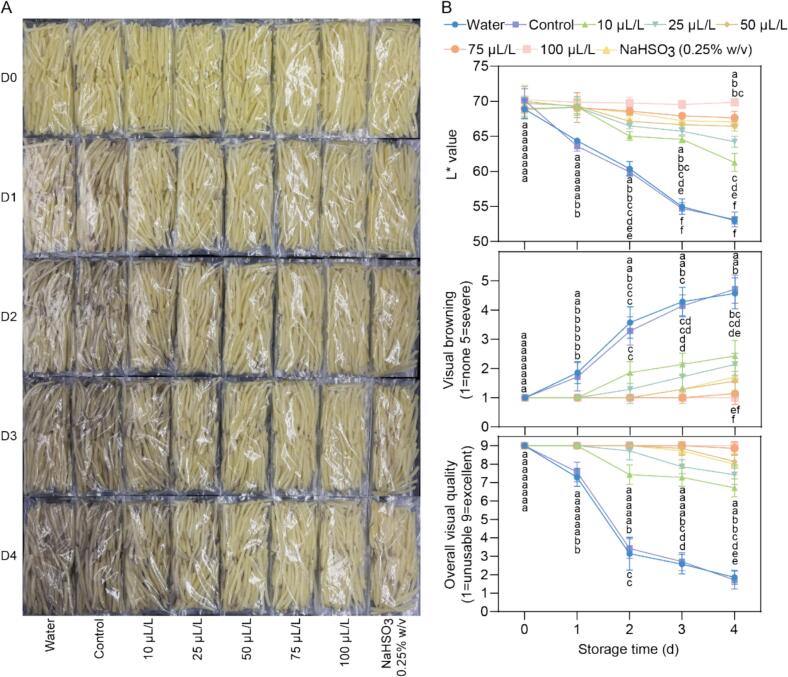
Effects of EMP on Browning and Quality of Fresh-Cut Potatoes During Storage. (A) Representative images of fresh-cut potato samples treated with different concentrations of EMP (0, 10, 25, 50, 75, and 100 μL/L), a water control, a negative control(no treatment), and a positive control (NaHSO_3_) during 4 days of storage at 4 °C. D0, D1, D2, D3, and D4 represent storage time points of 0, 1, 2, 3, and 4 days, respectively. (B) Changes in L* value (lightness), visual browning score (1 = none, 5 = severe), and overall visual quality score (1 = unusable, 9 = excellent) of fresh-cut potato samples over the 4-day storage period. Data points represent the mean ± standard deviation (*n* = 3). Different lowercase letters (a-f) within each time point indicate statistically significant differences among treatments according to Tukey's HSD test (*p* < 0.05).

The effect of EMP treatment on enzymatic browning in fresh-cut potatoes was assessed visually and quantitatively over a 4-day storage period (Fig. 1). Untreated control samples (both water and negative control) exhibited progressive browning over the 4 days, as evidenced by the darkening of the potato pieces (Fig. 1A). Conversely, samples treated with EMP displayed a concentration-dependent reduction in browning (Fig. 1A). Notably, the 50 μL/L, 75 μL/L, and 100 μL/L EMP treatments exhibited the most pronounced anti-browning effects, comparable to the positive control treated with a standard browning inhibitor, sodium bisulfite (NaHSO_3_) (Fig. 1A). The L* value, a measure of lightness, decreased significantly in the control groups (water and no treatment) over time, indicating substantial darkening. All EMP treatments maintained higher L* values than the controls throughout the experimental period. From day 2 onward, the 50, 75, and 100 μL/L EMP treatments and NaHSO_3_ exhibited significantly higher L* values than the other groups, demonstrating effective browning inhibition. The visual browning scores corroborated the L* value trends. Control samples exhibited a substantial increase in browning scores, reaching severe levels by day 4. EMP treatments significantly reduced browning scores in a concentration-dependent manner. On days 3 and 4, the 50, 75, and 100 μL/L EMP treatments, along with NaHSO_3_, displayed the lowest browning scores. They were statistically similar to each other and showed no significant difference in browning between day 3 and day 4, suggesting sustained efficacy. Overall visual quality scores decreased for all samples over time. However, the decline was less pronounced in EMP-treated samples, particularly at concentrations of 50, 75, and 100 μL/L. These treatments, similar to NaHSO_3_, maintained significantly higher quality scores compared to the controls from day 2 onwards. No significant difference was observed between the 50, 75, and 100 μL/L EMP treatments and NaHSO_3_ from day 2 to day 4. Notably, on day 4, only the 50 μL/L, 75 μL/L, and 100 μL/L EMP treatments and the NaHSO_3_ treatment retained acceptable visual quality, whereas the other treatments resulted in unacceptable quality degradation.

### 3.2 EMP inhibits browning through direct enzyme inhibition, not pH modification

**Fig. 2:**
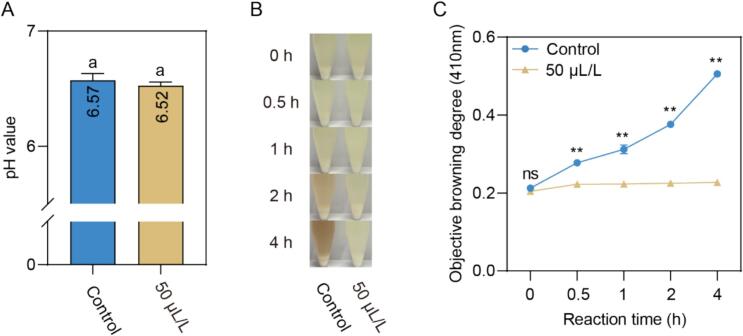
Effect of EMP on pH, visual appearance, and objective browning degree of potato pulp. **(A)** The pH values of solution with 50 μL/L EMP and control (0 μL/L EMP). Data are presented as mean ± standard deviation (*n* = 3). Different lowercase letters (a) indicate statistically significant differences between treatments according to the *t*-test (*p* < 0.05). **(B)** Images of potato pulp treated with 50 μL/L EMP and control at different time points (0, 0.5, 1, 2, and 4 h) during incubation at 25 °C. **(C)** Changes in absorbance at 410 nm, representing the objective browning degree, of potato pulp treated with 50 μL/L EMP and a control over a 4-h incubation period. Data points represent the mean ± standard deviation (n = 3). “ns” denotes no significant difference, and “**” indicates a significant difference between the two groups at *p* < 0.01, according to the Student's *t*-tests.

Building upon our previous findings demonstrating the potent anti-browning efficacy of 50 μL/L EMP treatment on fresh-cut potato slices (Fig. 1) and recognizing that the activity of potato PPO is sensitive to pH changes (Rasmussen et al., 2021), we examined the potential role of pH alteration in EMP's mechanism of action. As illustrated in Fig. 2A, no statistically significant difference in pH was observed between the control solution (0 μL/L EMP) and the 50 μL/L EMP solution. This result establishes that EMP does not exert its anti-browning effect through modifications of solution pH. A noticeable color difference was discernible, with the control sample exhibiting progressive browning over time, as indicated by a darkening of the pulp (Fig. 2B). In contrast, the EMP-treated sample maintained a lighter color, comparable to the initial appearance at 0 h, throughout the 4-h incubation period, signifying effective inhibition of enzymatic browning (Fig. 2B). To quantify the extent of browning inhibition, absorbance measurements at 410 nm were conducted (Fig. 2C). Higher absorbance values at this wavelength correspond to a greater degree of browning. The control sample exhibited a significant increase in absorbance over time, confirming the visual observation of progressive browning. Conversely, the EMP-treated sample showed significantly lower absorbance values than the control at all time points from 0.5 h onwards. While a slight increase in absorbance was observed in the EMP-treated sample at the 4-h mark, it remained significantly lower than the control at all time points, further substantiating the efficacy of EMP in inhibiting browning in potato pulp. These results demonstrate that 50 μL/L EMP treatment effectively inhibits the browning of potato pulp without significantly altering the pH. The visual and quantitative data strongly suggest that EMP's anti-browning activity is likely attributable to mechanisms other than pH modification, such as direct inhibition of polyphenol oxidase (PPO) activity. Consistent with its observed activity in potatoes, EMP also suppressed browning in tobacco leaf pulp, albeit necessitating higher concentrations (100 μL/L) for equivalent efficacy (Fig. S1). This observation suggests that EMP's anti-browning activity extends beyond a single plant species. Still, the optimal concentration is tissue-dependent, likely influenced by factors such as PPO abundance and activity, endogenous phenolic substrate profiles, and cellular architecture.

### 3.3 EMP suppresses PPO activity and quinone formation

To elucidate the mechanism underlying EMP's anti-browning effects, we investigated its impact on polyphenol oxidase (PPO) activity, copper chelation, and quinone formation in potato pulp. EMP treatment significantly suppressed PPO activity in potato pulp compared to the control. While the control group exhibited a substantial increase in PPO activity over the 4-h incubation period, the 50 μL/L EMP treatment inhibited PPO activity, maintaining it at near-zero levels throughout the experiment. Copper ions (Cu2+) are essential for PPO activity, as they are required at the enzyme's active site for its catalytic function. Chelating agents can bind to these copper ions, potentially reducing their availability to PPO and inhibiting its activity. While EMP treatment resulted in a higher copper chelating ability in potato pulp compared to the control, the difference did not reach statistical significance (*p* > 0.05). This suggests that although EMP might possess limited copper-chelating properties, this mechanism is unlikely to be the primary driver of the observed PPO inhibition. We further explore the effect of EMP on quinone content, a direct product of PPO-catalyzed oxidation. The control group showed a significant increase in quinone content over time, as indicated by the rising absorbance at 437 nm. In contrast, the 50 μL/L EMP treatment significantly inhibited quinone formation, maintaining quinone levels at significantly lower values than the control at all time points from 0.5 h onwards. These results indicate that EMP significantly inhibits PPO activity and subsequent quinone formation in potato pulp, with direct enzyme inhibition likely playing a more substantial role than copper chelation.

### 3.4 Purified PPO shows nanomolar sensitivity to EMP inhibition

To confirm the direct interaction between EMP and PPO, we purified potato PPO and assessed EMP's in vitro inhibitory effect on the purified enzyme's activity (Fig. 4). SDS-PAGE analysis of fractions obtained during the purification process is shown in Fig. 4A. The crude extract (Lane 2) and the supernatant after centrifugation (Lane 3) exhibited numerous protein bands, reflecting the complexity of the initial protein mixture. The flow-through (Lane 4) and wash fractions (Lane 5) showed reduced protein bands, indicating the removal of unbound or weakly bound proteins. The eluted fraction (Lane 6) displayed a prominent band at approximately 72 kDa, consistent with the predicate molecular weight, indicating successful enrichment of PPO.

The effect of EMP on the activity of the purified PPO was investigated (Fig. 4). Increasing concentrations of EMP resulted in a concentration-dependent decrease in PPO activity, demonstrating an apparent inhibitory effect. The data fits a typical dose-response curve. The IC_50_ value, calculated to be 156.7 ± 17.26 nM, indicates that EMP is a potent inhibitor of purified potato PPO, requiring only nanomolar concentrations to achieve 50 % inhibition of enzyme activity. These results strongly support the hypothesis that direct inhibition of PPO activity is a major contributing factor to the anti-browning effects of EMP.

### 3.5 Molecular modeling reveals competitive binding of EMP at the PPO active site

To elucidate the competitive inhibition mechanism of EMP against potato PPO concerning its preferred substrate, 4-methyl catechol (4-MC) (Rasmussen et al., 2021), computational modeling was employed (Fig. 5, Fig. S2). Given the absence of a crystal structure for potato PPO, these models provide a valuable, albeit theoretical, framework for understanding EMP's inhibitory action. The predicted structure of the potato PPO active domain in complex with 4-MC reveals key interactions within the active site (Fig. 5A). The 2D interaction diagram indicates that 4-MC interacts with residues His56, His74, His204, His208, Phe233, and Ala236 (Fig. 5A). The predicted binding mode of EMP suggests competitive inhibition of PPO (Fig. 5B). EMP occupies the active pocket, engaging residues His56, His74, His83, His204, Gly205, His208, His246, and Phe233 (Fig. 5B). Critically, the sulfur atom of EMP directly interacts with the two copper ions, Cu-1 and Cu-2, which are essential for PPO's catalytic activity (Fig. 5B). These findings suggest that EMP competitively inhibits PPO by occupying the active pocket and interfering with the copper cofactor. Analysis of a model with both 4-MC and EMP present provides further evidence for competitive inhibition (Fig. 5C). While EMP maintains interactions with crucial active site residues His56, His74, His83, His204, His208, and Phe233, 4-MC is displaced mainly from the active pocket (Fig. 5C). In this co-bound state, 4-MC primarily interacts with residues Ala236, Gly231, and Gly205, located further from the catalytic center (Fig. 5C). This displacement of 4-MC and the continued binding of EMP to key active site residues strongly suggest that EMP competitively inhibits PPO by preventing 4-MC from accessing its optimal binding site.

### 3.6 MD simulations demonstrate EMP-induced structural rigidification of PPO

To investigate the structural dynamics and stability of the potato PPO in complex with its substrate 4-MC and the ternary complex with both 4-MC and the inhibitor EMP, we performed 100 ns molecular dynamics (MD) simulations. The Root Mean Square Deviation (RMSD) of the protein backbone atoms was calculated relative to the initial structure to assess the overall structural stability of the complexes (Fig. 6A). The PPO-4-MC complex (blue line) exhibits a rapid increase in RMSD within the first 10 ns, reaching a value of approximately 0.35 nm, after which it fluctuates around a relatively stable plateau, indicating that the system has reached equilibrium. The PPO-4-MC-EMP complex (red line) shows an extremely low RMSD (around 0.05 nm) throughout the simulation, indicating high stability and minimal conformational change from the initial structure. The considerably lower RMSD of the ternary complex suggests that the presence of EMP significantly restricts the conformational flexibility of PPO. The radius of gyration (Rg) measures the protein's compactness (Fig. 6B). The PPO-4-MC complex shows a slight decrease from 1.98 nm to 1.95 nm and then small fluctuations in Rg over the simulation, with an average value of approximately 1.95 nm, suggesting that the overall compactness of the complex remained relatively stable. The PPO-4-MC-EMP complex maintains a consistently lower Rg value of around 1.87 nm, indicating a more compact structure than the binary PPO-4-MC complex. This further supports the notion that EMP binding induces a more compact and rigid conformation of PPO. The solvent-accessible surface area (SASA) reflects the extent to which the protein's surface is exposed to the solvent (Fig. 6C). The PPO-4-MC complex exhibits an average SASA of approximately 185 nm^2^, with some fluctuations. The PPO-4-MC-EMP complex displays a significantly lower average SASA of roughly 165 nm^2^, with more minor fluctuations. This decrease in SASA upon EMP binding is consistent with the observed reduction in Rg, indicating a more compact and less solvent-exposed structure in the presence of the inhibitor. The root mean square fluctuation (RMSF) was calculated to analyze the flexibility of individual residues (Fig. 6D). The PPO-4-MC complex shows significant fluctuations in several regions, particularly around residues 75–100, 150–175, and 225–275, indicating higher flexibility in these loop regions. In contrast, the PPO-4-MC-EMP complex exhibits drastically reduced RMSF values across almost all residues, indicating a significant reduction in residue-level flexibility. This demonstrates that EMP binding rigidifies the overall protein structure, not just the active pocket, locking the protein into a less dynamic conformation. The regions of high RMSF in the PPO-4-MC complex likely correspond to flexible loops stabilized by EMP binding in the ternary complex. In summary, the MD simulations reveal significant differences in the structural dynamics of PPO when bound to 4-MC alone versus when bound to both 4-MC and EMP. The presence of EMP leads to a more stable, compact, and less flexible PPO structure, as evidenced by lower RMSD, Rg, SASA, and RMSF values. These findings provide structural insights into the mechanism of EMP inhibition, suggesting that it restricts the conformational flexibility of PPO, potentially hindering substrate access or catalysis.

### 3.7 EMP reduces 4-MC binding affinity and disrupts substrate-enzyme interactions

To further elucidate the influence of EMP on the interaction between potato PPO and its substrate 4-MC, we analyzed the distance between the two molecules, the number of close contacts, and the binding free energy components from the 100 ns MD simulations. The distance between the centers of mass of PPO and 4-MC fluctuated around an average of 0.47 nm in the PPO-4-MC complex, indicating a relatively stable binding pose. In contrast, a significantly more considerable distance, with an average of 1.22 nm, was observed in the PPO-4-MC-EMP complex. This substantial increase in the distance suggests that 4-MC is positioned further away from the PPO active pocket in the presence of EMP, indicating a partial displacement. A relatively high number of close contacts (average of 8.04 PPO residue pairs within 0.35 nm of 4-MC) was maintained throughout the simulation in the PPO-4-MC complex. The PPO-4-MC-EMP complex exhibited a significantly reduced number of close contacts between PPO and 4-MC (average of 6.69 pairs), compared to the complex with 4-MC alone. This reduction further supports the conclusion that EMP weakens the interaction between PPO and 4-MC.

Binding free energy decomposition analysis was performed to quantify the energetic contributions to 4-MC binding in both the presence and absence of EMP (Fig. 7E). The total binding free energy (ΔTOTAL) for the PPO-4-MC complex is −19.11 kcal/mol, indicating a favorable interaction. In the PPO-4-MC-EMP complex, the ΔTOTAL is −14.48 kcal/mol, a significantly less favorable value, indicates that EMP reduces the binding affinity of 4-MC to PPO. The primary reason for the less favorable ΔTOTAL in the PPO-4-MC-EMP complex is reflected in the more positive ΔEGB and ΔGSOLV. In summary, the MD simulation results show that the presence of EMP significantly impacts the structural dynamics and binding to the PPO active site. The inhibitor shifts the 4-MC further away from the center of mass of the PPO, decreases its binding free energy, and lessens the number of close PPO residue contacts.

### 3.8 Dynamic evolution of PPO-ligand interactions

Time-resolved analysis of PPO-ligand interactions during 100 ns MD simulations revealed distinct binding dynamics between binary and ternary complexes (Fig. 8). In the PPO-4-MC complex, the substrate maintained stable interactions with key residues His56, His83, His246, and Tyr86 throughout the simulation, establishing a consistent binding mode characteristic of productive enzyme-substrate complexes.

The ternary PPO-4-MC-EMP complex exhibited markedly different interaction profiles. At 1 ns, EMP rapidly occupied critical active site residues His56, His83, and His246—the same residues essential for 4-MC binding in the binary complex. This immediate competition for binding sites displaced 4-MC from its optimal catalytic position, as evidenced by the altered 2D interaction patterns compared to the binary complex.

Temporal evolution analysis revealed EMP's persistent engagement with copper-coordinating histidines, maintaining these crucial interactions even in the presence of 4-MC. This sustained occupancy of metal coordination sites prevents proper substrate positioning for electron transfer to the copper centers, mechanistically explaining the competitive inhibition. The dynamic data demonstrate that EMP's inhibitory effect results from both direct competition for binding sites and sustained disruption of the catalytic geometry required for phenolic oxidation.

## 4 Discussion

This study establishes ethyl 3-mercaptopropionate (EMP) as a highly effective and safe anti-browning agent for fresh-cut potatoes, demonstrating its potential as a novel alternative to conventional inhibitors like sulfites. The results reveal that EMP, at concentrations of 50–100 μL/L, significantly mitigates enzymatic browning and preserves the visual quality of fresh-cut potato slices over a 4-day storage period at 4 °C, performing comparably to sodium bisulfite (NaHSO_3_), a widely used commercial standard (Fig. 1). The concentration-dependent inhibition observed in both visual assessments and quantitative metrics—such as L* values, browning scores, and overall quality scores—underscores EMP's practical utility in maintaining the aesthetic and market appeal of fresh-cut produce. Given EMP's established safety as a food flavoring agent, recognized by the European Union (EU) and the Joint FAO/WHO Expert Committee on Food Additives (JECFA), its adoption could address consumer and regulatory concerns over sulfite-related health risks, such as allergic reactions in sensitive populations (Iyengar & McEvily, 1992).

**Fig. 3:**
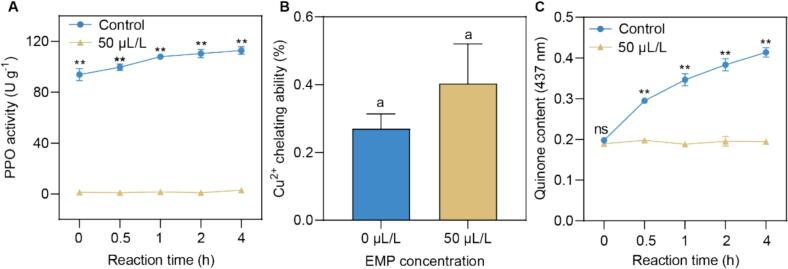
Effect of EMP on PPO Activity, Copper Chelating Ability, and Quinone Content in Potato Pulp. **(A)** Changes in PPO activity (U g < sup> − 1</sup>) in potato pulp treated with 50 μL/L EMP and a control over a 4-h incubation period. **(B)** Copper Chelating Ability (%) of potato pulp treated with 0 μL/L (control) and 50 μL/L EMP. **(C)** Changes in quinone content, measured by absorbance at 437 nm, in potato pulp treated with 50 μL/L EMP and control over a 4-h incubation period. Data are presented as mean ± standard deviation (*n* = 3). “ns” denotes no significant difference, and “**” indicates a significant difference between the control and EMP treatment groups at *p* < 0.01, according to the student *t*-test. Different lowercase letters (a) in (B) indicate a statistically significant difference between treatments (*p* < 0.05).

The mechanistic basis of EMP's anti-browning efficacy was explored through its effects on potato pulp and purified polyphenol oxidase (PPO). Unlike pH-altering agents, EMP does not significantly modify the pH of treatment solutions (Fig. 2A), ruling out indirect effects via pH-dependent PPO inactivation (Rasmussen et al., 2021). Instead, its potent inhibition of browning in potato pulp, as evidenced by reduced absorbance at 410 nm (Fig. 2C), indicates a direct interaction with PPO. This was substantiated by enzyme activity assays showing near-complete suppression of PPO activity and quinone formation at 50 μL/L EMP over a 4-h incubation (Fig. 3A, C). The slight quinone increase observed after 4 h in EMP-treated samples may reflect minor contributions from non-enzymatic oxidation pathways or residual PPO activity, a phenomenon noted in recent studies of PPO inhibitors (Sui et al., 2023). Although EMP exhibited a modest, non-significant increase in copper chelating ability (Fig. 3B), this effect appears secondary to its primary mode of action. Copper, a critical cofactor in PPO's active site (Tilley et al., 2023), is a plausible target for sulfur-containing compounds like EMP, yet the data suggest that direct enzyme inhibition predominantly drives its efficacy.

The mechanism of EMP aligns with recent findings on sulfur-containing polyphenol oxidase (PPO) inhibitors. S-Ethyl thioacetate, a natural anti-browning agent, reduces PPO activity through direct interaction with enzyme residues (Feng, Sun, et al., 2022), similar to EMP's proposed action. Additionally, 3-mercapto-2-butanol functions as a competitive inhibitor that suppresses PPO activity and decreases tyrosine consumption (Ru et al., 2020). The thiol group present in EMP and these related compounds appears central to their inhibitory capacity, as sulfur-containing moieties can disrupt the copper-dependent active site essential for PPO function. These findings collectively support the hypothesis that EMP's anti-browning efficacy stems from sulfur-mediated interference with the enzyme's catalytic center.

**Fig. 4:**
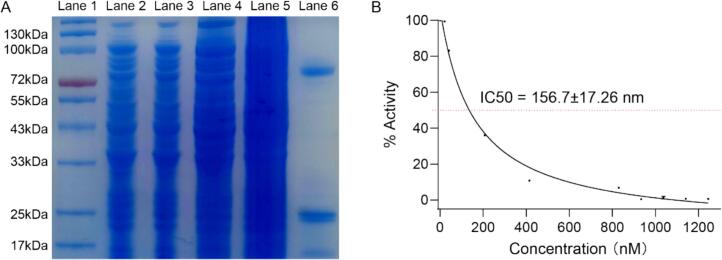
Purification of PPO and Determination of its Inhibition by EMP. (A) SDS-PAGE Analysis of Potato PPO Purification. Lane 1: Molecular weight marker. Lane 2: Crude potato extract. Lane 3: Supernatant after centrifugation. Lane 4: Flow-through from the purification column. Lane 5: Wash fraction from the purification column. Lane 6: Eluted fraction containing purified potato PPO. The gel was stained with Coomassie Brilliant Blue. The expected molecular weight of potato PPO is around 72 kDa. (B) Inhibitory Effect of EMP on Purified Potato PPO Activity. The graph shows the percentage of remaining PPO activity plotted against increasing concentrations of EMP (nM). The IC_50_ value, representing the concentration of EMP required to inhibit 50 % of PPO activity, was determined to be 156.7 ± 17.26 nM. Data are presented as mean ± standard deviation (*n* = 3). (For interpretation of the references to color in this figure legend, the reader is referred to the web version of this article.)

The purification of potato PPO and subsequent kinetic analysis further confirmed EMP's potency, with an IC₅₀ of 156.7 ± 17.26 nM against the purified enzyme (Fig. 4B). This nanomolar-range potency aligns with the observed anti-browning effects in whole-tissue and pulp assays. The thiol group in EMP, a structural feature shared with known PPO inhibitors like cysteine (Zhang et al., 2023), likely underpins its inhibitory capacity. Recent research has shown that sulfur-containing compounds disrupt PPO activity by interacting with copper ions or active site residues (Wang et al., 2023), a hypothesis explored further through molecular modeling.

**Fig. 5:**
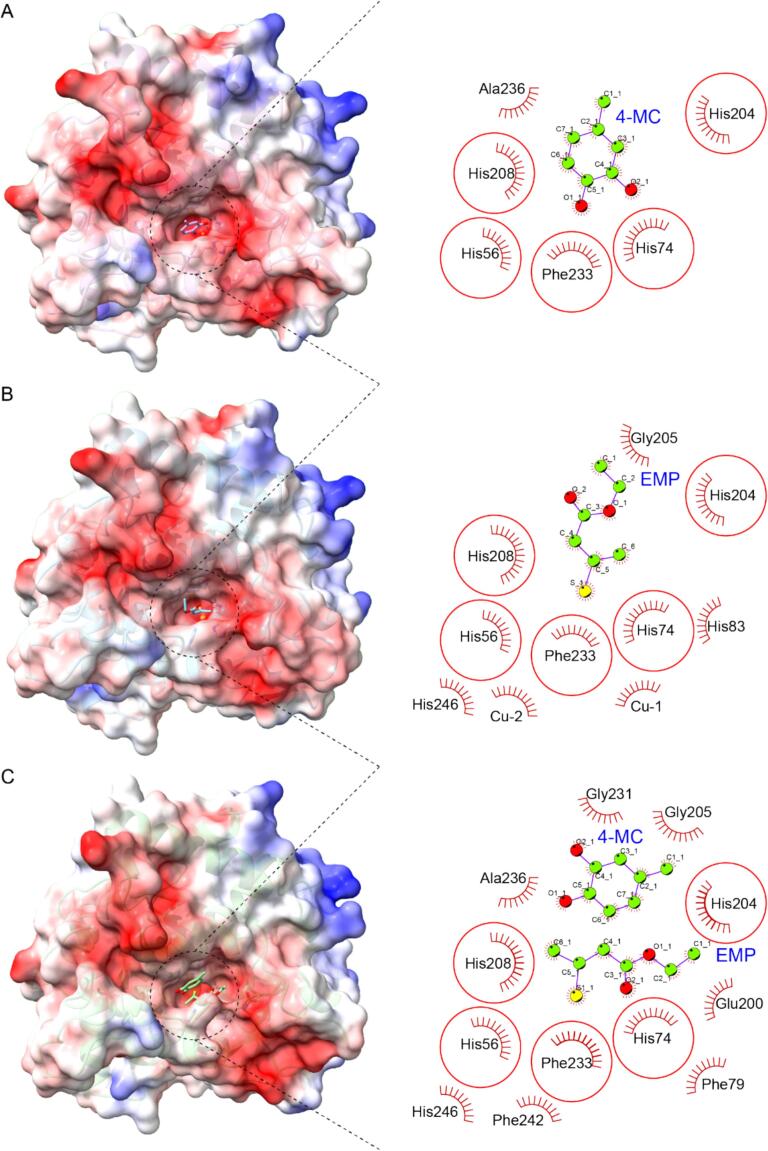
Predicted Potato PPO Active Domain structure and its Interaction with Substrate 4-MC and Inhibitor EMP. (A) Predicted Structure of the PPO Active Domain with Bound 4-MC. (B) Predicted Structure of the PPO Active Domain with Bound EMP. (C) Predicted Structure of the PPO Active Domain with Bound 4-MC and EMP. The surface representation of the predicted PPO active domain is colored by electrostatic potential (red: negative, blue: positive). Key residues involved in interactions are labeled. (For interpretation of the references to color in this figure legend, the reader is referred to the web version of this article.)

**Fig. 6:**
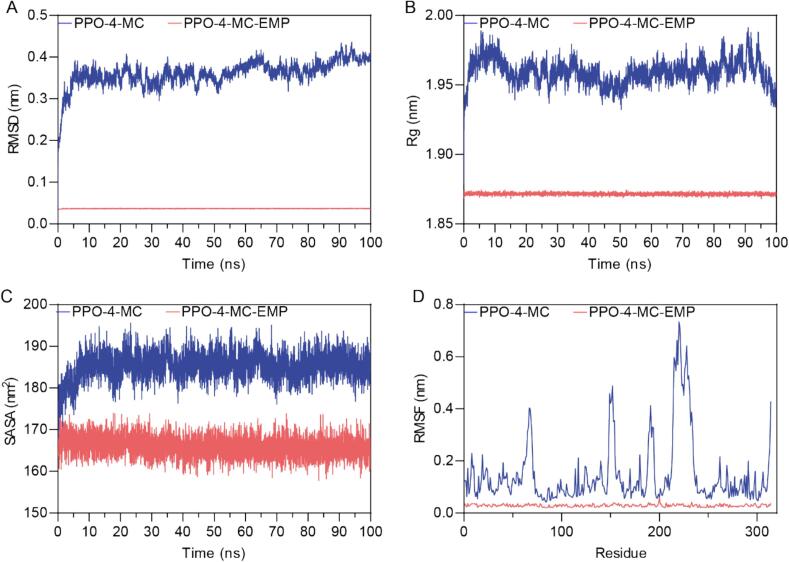
Molecular Dynamics Simulation Analysis of PPO-4-MC and PPO-4-MC-EMP. (A) Root Mean Square Deviation (RMSD) of the protein backbone atoms over the 100 ns simulation time. (B) Radius of Gyration (Rg) of the protein over the 100 ns simulation time. (C) Solvent Accessible Surface Area (SASA) of the protein over the 100 ns simulation time. (D) Root Mean Square Fluctuation (RMSF) of individual protein residues.

**Fig. 7:**
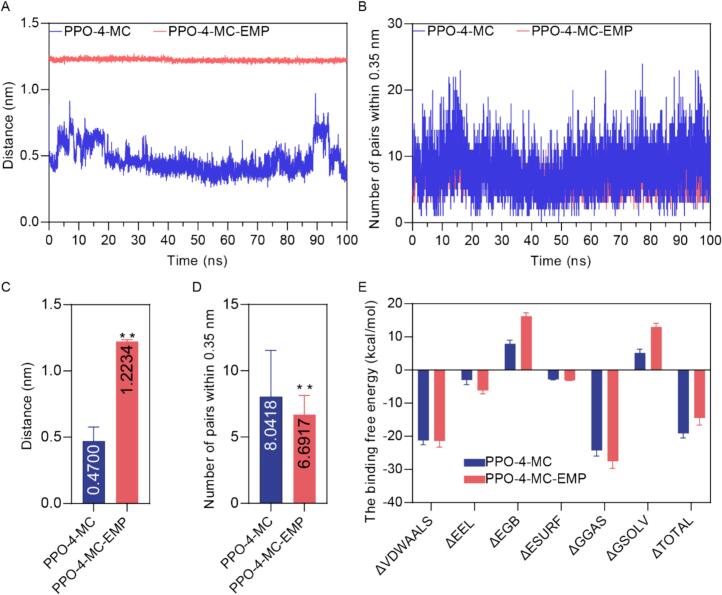
Analysis of 4-MC Interactions with Potato PPO in the Presence and Absence of EMP from 100 ns Molecular Dynamics Simulations. (A) Time evolution of the distance between the centers of mass of PPO and 4-MC. (B) Number of PPO residue pairs within 0.35 nm of 4-MC over the 100 ns simulation time. (C) The average distance between the centers of mass of PPO and 4-MC is calculated from the data in panel A. Error bars represent the standard deviation. (D) The average number of PPO residue pairs within 0.35 nm of 4-MC, calculated from the data in panel B. Error bars represent the standard deviation. Asterisks (**) indicate a statistically significant difference (*p* < 0.01) between the two complexes. (E) Decomposition of the binding free energy (kcal/mol) between PPO and 4-MC into different energy components: ΔVDWAALS (van der Waals interactions), ΔEEL (electrostatic interactions), ΔEGB (polar solvation energy), ΔESURF (nonpolar solvation energy), ΔGGAS (gas-phase interaction energy), ΔGSOLV (solvation free energy), and ΔTOTAL (total binding free energy). Error bars represent the standard deviation.

Computational analyses, including molecular modeling and 100 ns molecular dynamics (MD) simulations, provided atomic-level insights into EMP's competitive inhibition of PPO. In the absence of an experimental crystal structure for potato PPO, the predicted active domain structure revealed that EMP binds the enzyme's active pocket, overlapping with the substrate 4-methylcatechol (4-MC) binding site (Fig. 5). EMP's sulfur atom interacts directly with the catalytic copper ions (Cu-1 and Cu-2) and key histidine residues (e.g., His56, His74, His204, His208), which coordinate these cofactors (Fig. 5B). This binding displaces 4-MC from its optimal catalytic position in the ternary PPO-4-MC-EMP complex (Fig. 5C), reducing its access to the active site. MD simulations reinforced this mechanism, showing that EMP stabilizes PPO into a more compact, rigid conformation, as evidenced by lower RMSD, radius of gyration (Rg), solvent-accessible surface area (SASA), and residue-level RMSF values compared to the PPO-4-MC complex (Fig. 6). This structural rigidification likely restricts the conformational flexibility required for substrate binding and catalysis (Rasmussen et al., 2021). Binding free energy analysis further quantified EMP's impact, revealing a less favorable ΔG_TOTAL for 4-MC in the presence of EMP (−14.48 kcal/mol vs. -19.11 kcal/mol in the binary complex; Fig. 7E), driven by increased polar solvation penalties (ΔE_GB) that destabilize 4-MC binding.

**Fig. 8:**
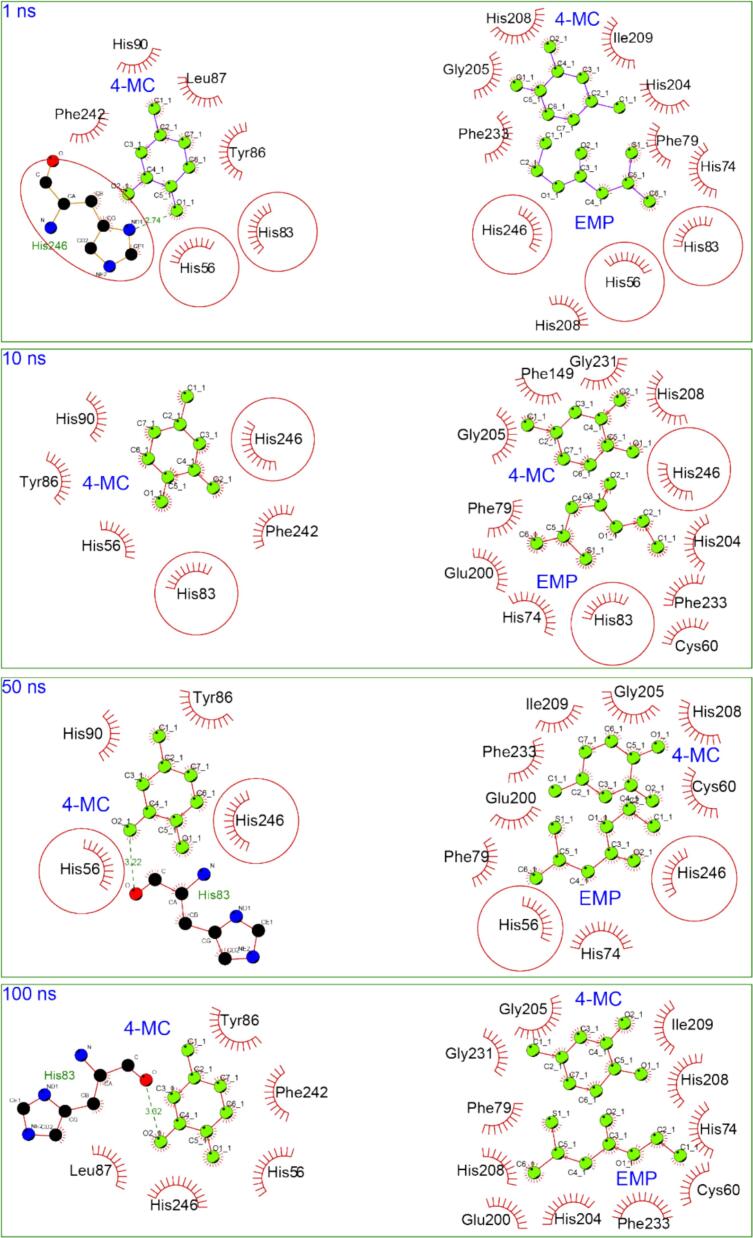
Dynamic Interactions of Potato PPO-ligand complexes at Different Time Intervals during a 100 ns Molecular Dynamics Simulation. Circles represent residues that participate in interactions with 4-MC in the binary PPO-4-MC complex and are also involved in interactions with EMP in the ternary PPO-4-MC-EMP complex, highlighting residues with shared binding roles.

The 2D interaction diagrams reveal a progressive binding stabilization mechanism where EMP establishes increasingly favorable contacts over the simulation timeframe (Fig. 8). The temporal evolution demonstrates kinetic selectivity where EMP's binding affinity increases with residence time, while 4-MC exhibits weakening interactions due to geometric strain. This competitive displacement aligns with classical models of enzyme inhibition and mirrors the behavior of other sulfur-containing PPO inhibitors, such as those derived from Allium species (Arnold & Gramza-Michałowska, 2022; Bernaś & Jaworska, 2021; Chauhan & Semwal, 2024).

The mechanistic insights revealed in our study align with recent findings on sulfur-containing PPO inhibitors. Several sulfur compounds, including sodium metabisulfite, dithiothreitol, sodium dithionite, and cysteine, effectively inhibit PPO activity in various plant tissues (Zhang, 2023). The thiol moiety in EMP appears crucial for inhibition, similar to other effective inhibitors. Binding to secondary sites on PPO can lead to conformational changes that affect catalytic efficiency (Lim et al., 2012), consistent with our observations of EMP-induced structural rigidification.

The broader implications of this study are significant for the food industry, where enzymatic browning remains a persistent challenge in fresh-cut produce processing (Shevkani, 2024). EMP's dual advantages—safety as a flavoring agent and potency as a PPO inhibitor—position it as a viable alternative to sulfites and synthetic agents, aligning with consumer demand for natural, minimally processed foods (Navina et al., 2023). Its efficacy extends beyond potatoes, as demonstrated by browning inhibition in tobacco leaf pulp (Fig. S1), albeit at higher concentrations (100 μL/L), suggesting tissue-specific optimization may be required due to differences in PPO isoforms, phenolic content, or cellular matrices (Tsikrika et al., 2022). This versatility enhances EMP's potential applicability to other browning-susceptible crops, such as apples (Zou et al., 2025) or mushrooms (Wang et al., 2025).

Our evaluation extended beyond anti-browning efficacy to assess EMP's impact on fresh-cut potato quality attributes. As demonstrated in Fig. S3, EMP treatment (50 μL/L) maintained comparable dry matter content, soluble solids, and textural hardness to those of the control and sodium bisulfite treatments, indicating preservation of fundamental physical properties. While these results suggest EMP maintains critical quality parameters, future research should address its effects on sensory perception, a crucial factor for consumer acceptance (Cui et al., 2024; Jang & Lee, 2024). Given EMP's natural occurrence as a flavor component in foods like Concord grapes and ripened cheeses, its potential to impart subtle aromatic notes warrants formal sensory evaluation.

The simulation conditions (300 K, 1 bar) differ from experimental storage conditions (4 °C), potentially affecting molecular interaction dynamics. Additionally, the simulations were conducted at neutral pH, whereas damaged plant tissues often experience pH fluctuations that could alter EMP-PPO interactions. Despite these limitations, the strong correlation between our computational predictions and experimental observations, particularly the nanomolar IC₅₀ value and quinone formation inhibition, supports the proposed competitive inhibition mechanism. Future research should leverage high-resolution structural techniques (e.g., X-ray crystallography or cryo-EM) to validate the predicted PPO-EMP interactions. These complementary approaches would bridge the gap between in silico models and experimental reality while facilitating the rational design of improved anti-browning agents.

## 5 Conclusions

This study elucidates the molecular mechanism by which ethyl 3-mercaptopropionate (EMP) functions as a competitive polyphenol oxidase (PPO) inhibitor through direct sulfur‑copper coordination and allosteric protein rigidification. The exceptional nanomolar potency (IC₅₀ = 156.7 nM) stems from EMP's thiol group forming coordinate bonds with catalytic copper ions while simultaneously occupying critical histidine residues (His56, His74, His204, His208) essential for substrate positioning and catalysis. Molecular dynamics simulations reveal that EMP binding induces protein rigidification (RMSD reduction from 0.35 to 0.05 nm) and creates an energetically unfavorable environment for substrate access (ΔG reduction of 4.63 kcal/mol). This dual mechanism—direct active site occupation combined with allosteric structural constraints—distinguishes EMP from conventional PPO inhibitors and explains its superior efficacy at low concentrations. The practical significance extends beyond browning control, as EMP's dual functionality as a GRAS-approved flavoring agent and potent enzyme inhibitor addresses the food industry's critical need for natural, multifunctional additives that eliminate sulfite-associated health concerns. The mechanistic insights provide a rational framework for designing next-generation sulfur-containing PPO inhibitors targeting copper-dependent enzymes, with potential applications extending to other oxidative enzymes in food preservation and biotechnology. These findings position EMP as a paradigm for developing safe and effective alternatives to synthetic preservatives, while advancing our understanding of thiol-mediated enzyme inhibition mechanisms.

## CRediT authorship contribution statement

**Guangcan Cui:** Validation, Software, Methodology, Investigation, Data curation. **Juncang Peng:** Software, Methodology, Investigation, Formal analysis, Data curation, Conceptualization. **PingPing Liu:** Validation, Methodology, Data curation. **Yonghong Wang:** Visualization, Formal analysis. **Chang Ge:** Software, Methodology. **Xiaoyong Chang:** Software, Methodology, Data curation. **Xueao Zheng:** Visualization, Formal analysis, Data curation. **Chen Wang:** Validation, Data curation. **Yalong Xu:** Software, Data curation. **Xiaozhan Qu:** Validation, Software. **Yixiao Zhang:** Validation, Formal analysis. **Peijian Cao:** Project administration. **Tengfei Liu:** Writing – review & editing, Writing – original draft, Visualization, Validation, Supervision, Investigation, Data curation, Conceptualization. **Qiansi Chen:** Writing – review & editing, Supervision, Project administration, Funding acquisition, Conceptualization.

## Declaration of competing interest

The authors declare that they have no known competing financial interests or personal relationships that could have appeared to influence the work reported in this paper.

## Data Availability

Data will be made available on request.

## Appendix A Supplementary data

Supplementary material Image 1
