# Supplementary material for: Ethyl 3-mercaptopropionate, a safe food flavoring, competitively inhibits polyphenol oxidase and prevents browning in fresh-cut produce
Source: Food Chem X. 2025 Aug 2;29:102859. doi: 10.1016/j.fochx.2025.102859 (PMC12341628; doi:10.1016/j.fochx.2025.102859)
Supplement: Supplementary file 1 — Supplementary material [file mmc1.docx]

**Ethyl 3-Mercaptopropionate, a Safe Food Flavoring, Competitively Inhibits Polyphenol Oxidase and Prevents Browning in Fresh-Cut Produce**

Guangcan Cui^1, 2#^, Juncang Peng^3#^, PingPing Liu^1^, Yonghong Wang ^3^, Chang Ge^1, 2^, Xiaoyong Chang^2^, Xueao Zheng^1^, Chen Wang^1^, Yalong Xu^1^, Xiaozhan Qu^1^, Yixiao Zhang ^1^, Peijian Cao^1, 4^, Tengfei Liu^2*^, Qiansi Chen^1, 4*^

^1^ Zhengzhou Tobacco Research Institute of CNTC, No. 2 Fengyang Street, Zhengzhou, Henan Province 450001, China

^2^ College of Food Science and Engineering, Shandong Agricultural University, Tai’an 271018, Shandong, China

^3^ Technology Center, China Tobacco Shaanxi Industrial Co., Ltd., Xian 710065, China

^4^ Beijing Life Science Academy (BLSA), Beijing 102209, China

^*^ Corresponding author:

E-mail addresses: hzauzsmj@gmail.com and liutengfei@sdau.edu.cn (T. Liu), chen_qiansi@163.com (Q. Chen).

^#^ The authors equally contributed to this work.

**Table S1** Primers used in this research

| Primers | Sequence (5’-3') | Related experiment |
| --- | --- | --- |
| POT32F | GAGAACAGATTGGTGGATCCCTCTCATCTTGTAGTAAAGC | PPO expression |
| POT32R | TGGTGGTGGTGGTGCTCGAGTTAACAATCTGCAAGACTGA | PPO expression |


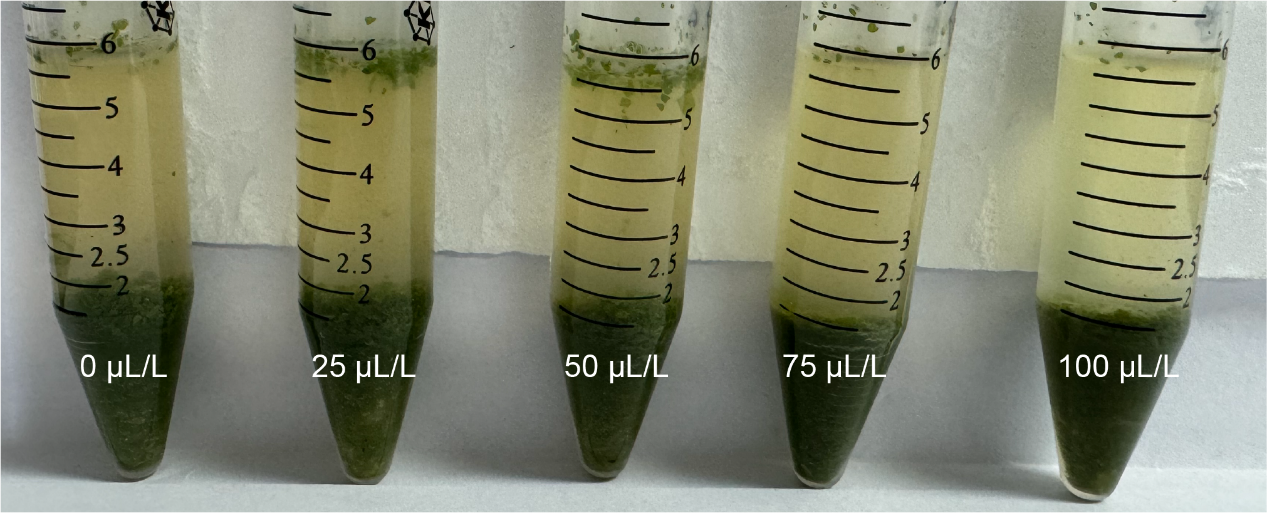


**Fig. S1** Impact of EMP on tobacco leaf pulp browning.


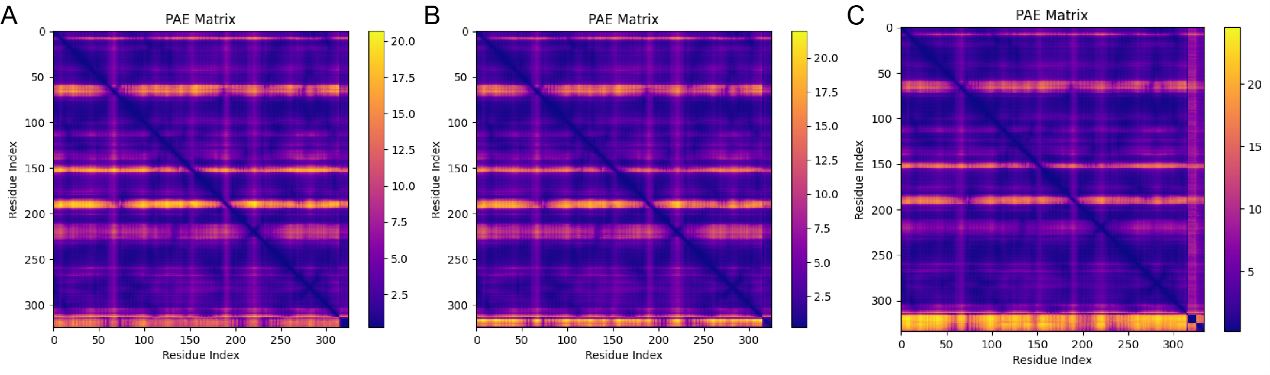


**Fig. S2** PAE matrix of PPO-ligand complexes prediction (darker is more confident). (A) PPO-4-MC complex. (B) PPO-EMP complex. (C) PPO-4-MC-EMP complex.


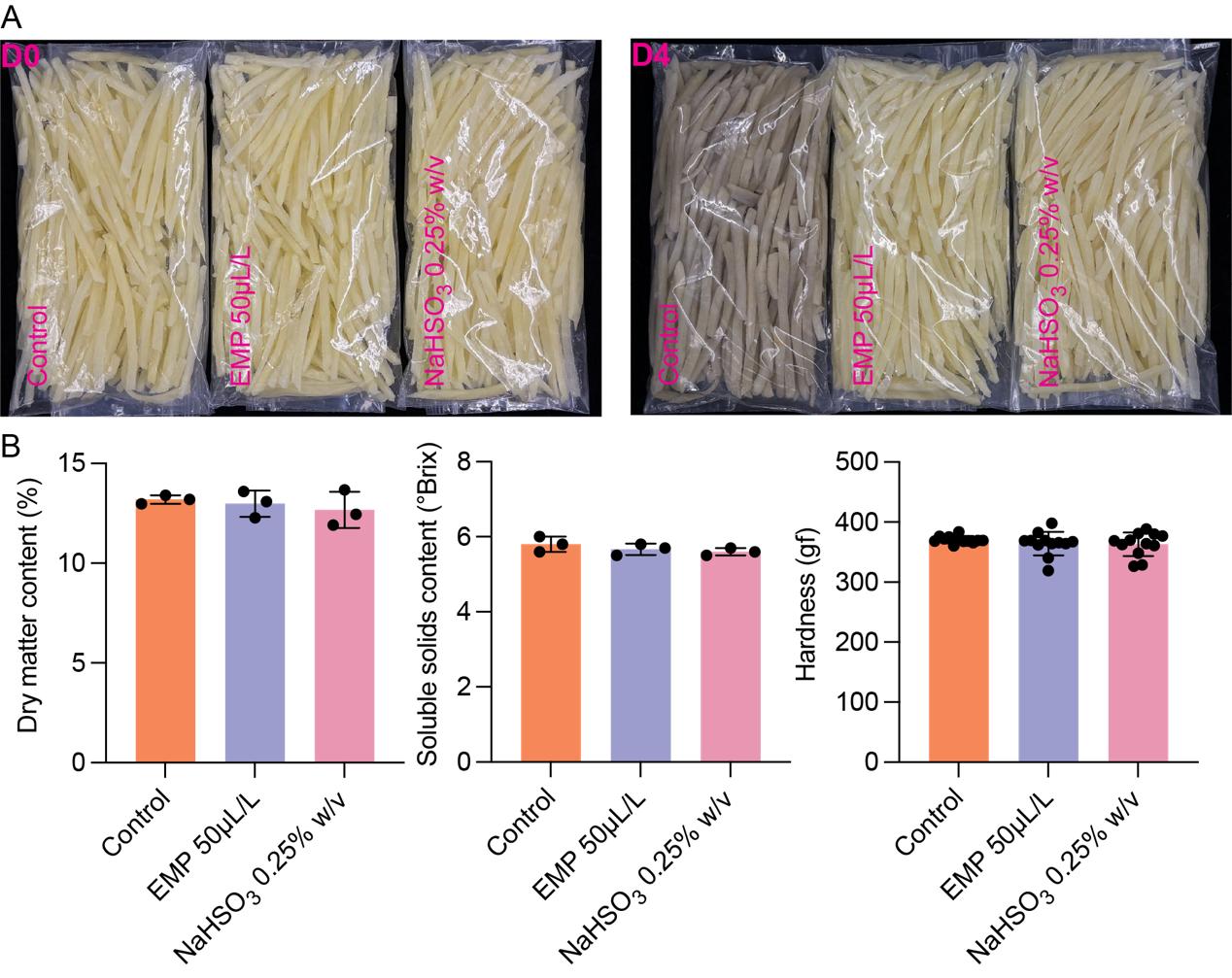


**Fig. S3** Effect of EMP treatment on quality parameters of fresh-cut potato. (A) Visual appearance of fresh-cut potato samples treated with control (water), EMP (50 µL/L), and NaHSO₃ (0.25% w/v) at day 0 (D0, left) and day 4 (D4, right) of storage at 4°C. (B) Quality parameters of fresh-cut potato samples after 4 days of storage, including dry matter content (%), soluble solids content (°Brix), and textural hardness (gf). Data for dry matter and soluble solids content are presented as mean ± standard deviation (n=3), while textural hardness data are presented as mean ± standard deviation (n=12). No significant differences were observed among treatments for any quality parameter (p > 0.05).
